# Integrated Bioinformatics Analysis for Target Identification and Evaluation of Recombinant Protein as an Antigen for Intradermal Skin Test in Bovine Tuberculosis Diagnosis

**DOI:** 10.1021/acsomega.4c09374

**Published:** 2025-02-24

**Authors:** Violetta Dias Pacce, Amanda Munari Guimarães, Frederico Schmitt Kremer, Gabriela Nascimento Ferreira, Jean Michel Dela Vedova-Costa, Aline Cristina dos Santos, Odir Antônio Dellagostin, Carlos Ricardo Soccol, Vanete Thomaz-Soccol

**Affiliations:** † Laboratório de Biologia Molecular, Programa de Pós Graduação em Engenharia de Bioprocessos e Biotecnologia, Universidade Federal do Paraná, Curitiba, Paraná 81531-990, Brazil; ‡ Programa de Pós Graduação em Biotecnologia, Centro de Desenvolvimento Tecnológico, Universidade Federal de Pelotas, Pelotas, Rio Grande do Sul 96160-000, Brazil; § Laboratório Provas Biológicas, Instituto de Tecnologia do Paraná, Curitiba, Paraná 80035-060, Brazil

## Abstract

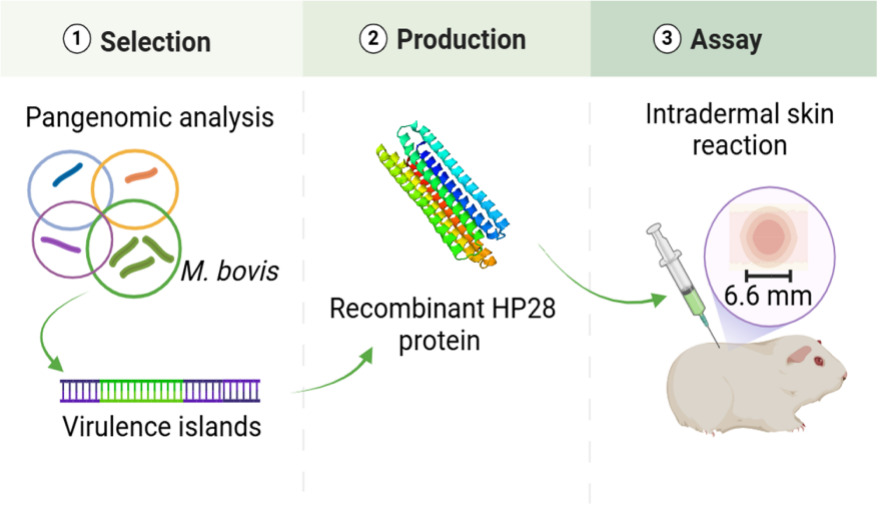

Bovine tuberculosis (bTB) is a respiratory disease caused by *Mycobacterium bovis*, posing a significant threat to animal health and the livestock industry. Current control strategies for bTB rely on diagnostic tests and slaughter policies. However, the limitations of existing diagnostic methods, which depend on PPD antigens, necessitate the exploration of alternative antigens to enhance the accuracy and reliability of bTB diagnosis. This study aimed to identify, produce, and evaluate novel antigens for use in the intradermal skin test for bTB diagnosis. A pangenome analysis of four *Mycobacterium* species identified 12 unique genes specific to *M. bovis* SP38. Further integrated bioinformatic analysis revealed 224 genomic islands associated with virulence and pathogenesis. Among these, a highly antigenic protein, termed HP28, was selected for in vivo testing. The recombinant HP28 protein (rHP28) was expressed in *E. coli* and assessed for its ability to induce intradermal skin reactions in guinea pigs. The rHP28 protein elicited a skin reaction of 6.6 mm at 72 h post-injection, whereas negative controls showed no reaction. This study presents a pipeline for the selection of antigens using integrated bioinformatic analysis to identify diagnostic targets that can effectively distinguish between sensitized and non-sensitized animals, offering a promising approach for improving bTB diagnostics.

## Introduction

*Mycobacterium bovis* is the causative agent of tuberculosis (TB) in animals, particularly in bovines. This bacterium is part of the *M. tuberculosis* Complex (MTC), characterized by slow evolution, strict clonality, and absence of gene transfer or recombination.^1^ However, despite having >99% nucleotide identity, MTC species have variable host tropisms, phenotypes, and pathogenic degrees.^2^ Clinical signs rarely manifest in animals, which often exhibit a healthy phenotype even if tested positive.^3^

Globally, bovine tuberculosis (bTB) causes an annual agricultural revenue loss of approximately 3 billion dollars^4,5^ owing to the loss of milk, meat, and carcass.^6^ The disease is also zoonotic, spreading to humans through close contact with infected cows or via the consumption of unpasteurized milk.^7^ A recent meta-analysis indicated that 702 (9.7%) of 7185 MTC isolates from human tuberculosis cases were caused by *M. bovis* subspecies.^8^ The disease burden is higher in marginalized populations and poor or rural communities living close to animals and lacking access to health care and/or safe food.^9,10^ For those reasons, the One Health concept has embraced the burden of bovine tuberculosis since the disease can only be abolished from humans when it is completely eradicated from animals as well. Routine diagnostic tests are necessary for better disease control and epidemiological monitoring. Currently, the intradermal skin test (TST) using Purified Protein Derivative (PPD) as an antigen is widely applied and recognized as a reference technique by the World Organization for Animal Health.^11^ However, bovine PPD can exhibit cross-reactivity due to the possible presence of proteins also found in nonpathogenic environmental mycobacteria. Additionally, the lack of antigen standardization can impact result consistency, and its production requires manipulation of a live virulent strain of *M. bovis*, raising biosafety concerns.^12,13^

Although several antigens such as ESAT-6, CFP-10, Rv3615c, and MPB70 are being explored for bovine tuberculosis diagnostics, their performance can be limited due to variability in sensitivity and specificity depending on test methodology used.^14^ In this scenario, the search for bTB diagnosis of new antigens has been ongoing, either to introduce a new methodology or to improve existing ones. The goal is to use antigens that are only present in pathogenic bacteria, which can differentiate between infected and healthy animals and/or vaccinated individuals. For new antigens’ search, pangenome analysis has proven effective in understanding the species’ characteristics to develop vaccines or diagnostic strategies.^15^ Moreover, the accumulation of biological data and computational advances in recent decades has made pangenome analysis a fast and concise method for genotype-phenotype evaluation and understanding.^16^

From this perspective, this study aimed to identify exclusive genes from virulence clusters that could be further explored as diagnostic targets for bovine tuberculosis control.

## Results

### Pangenome Analysis

**Figure 1:**
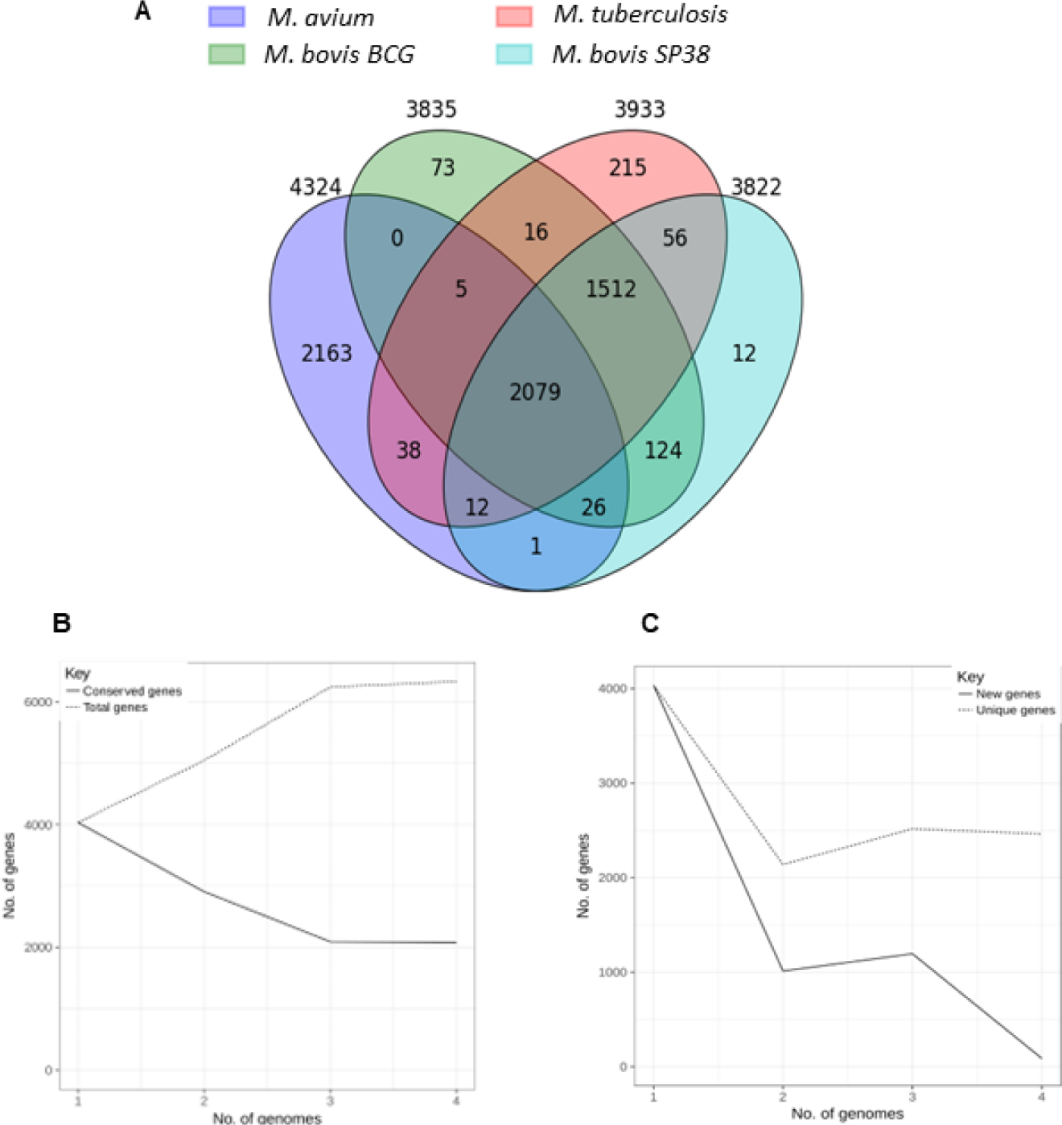
*Mycobacterium* pangenome. (A) Number of genes belonging to the core, the soft core, the shell or the cloud of the *Mycobacterium* strains; (B) continuous line (Conserved genes) shows how the number of conserved genes changes as more genomes are added to the analysis. In a pangenome, this line is expected to stabilize after a certain point, indicating that all essential or conserved genes have been identified. A horizontal continuous line suggests that these genes are highly conserved among the genomes. The dotted line (Total genes) illustrates how the total number of genes grows with the increasing number of genomes. Initially, the number of total genes rises rapidly because each new sequenced genome may contain unique genes not present in previous genomes. However, as more genomes are added, the new gene discovery rate decreases, and the line begins to stabilize. This indicates that most unique or variable genes have been identified. (C) Representation of the *Mycobacterium* gene content (extrapolated median-based line) according to how the pan-genome varies as genomes are added in random order to the analysis. The dotted line represents unique genes, whereas the continuous line represents new genes. The image of Figure 1A was generated through a custom Python script and graphics are derived from native output feature of Roary, which provides built-in visualizations of pangenome data.

Roary analysis of *Mycobacterium* strains pangenome identified 2079 core, 1555 soft core, 235 shell, and 2463 cloud genes, respectively, out of 6332 total genes (Figure 1A). The large number of cloud genes implies that a large heterogeneity exists among the four strains considered, highlighting the “open” nature of *Mycobacterium* pan-genome (Figure 1B,C).

### Genomic Island Analysis

For the genome *M. avium*, 32 genomic islands associated with virulence and pathogenesis were identified. As for *M. tuberculosis* H37Rv and *M. tuberculosis* variant *bovis,* 240 and 224 genomic islands related to virulence and pathogenesis were identified, respectively. Lastly, 159 islands were identified for the *M. tuberculosis* variant *bovis* BCG str. Pasteur. The respective islands for each organism can be found in Supplementary Material 1.

### Unique Protein Analysis and Antigenicity Evaluation

The Multiple Blast alignment for identities with less than 60% generated a single protein for *M. avium* (MAP01). For *M. bovis* BCG str. Pasteur, two proteins were found (MBCGP02 and MBCGP03). Alignment for *M. tuberculosis* H37Rv and *M. bovis* SP38 resulted in two proteins for both organisms, named MHR04, MHR05, MSP06, and MSP07, respectively. These proteins were classified as antigenic (≥ 0.5) or non-antigenic (< 0.5), following a 0.5 threshold (Table 1).

**Table 1:** Antigenicity Values for Unique Proteins Identified^a^

Protein	Organism	Antigenicity value
MAP01	*M. avium*	0.0657
MBCGP02	*M. bovis* BCG str. Pasteur	0.0747
MBCGP03	*M. bovis* BCG str. Pasteur	0.4330
MHR04	*M. tuberculosis* H37Rv	0.1982
MHR05	*M. tuberculosis* H37Rv	0.1278
MSP06	*M. bovis* SP38	0.3290
MSP07	*M. bovis* SP38	0.9272

a Antigenicity value is generated by ANTIGENPro software.

It is observed that protein MSP07 showed the highest antigenicity value, being classified as antigenic. This protein was termed by the authors “rHP28” protein, meaning Hypothetical Protein 28, with 28 representing the vector where it was further cloned into (pET28a+).

### Protein Structure

**Figure 2:**
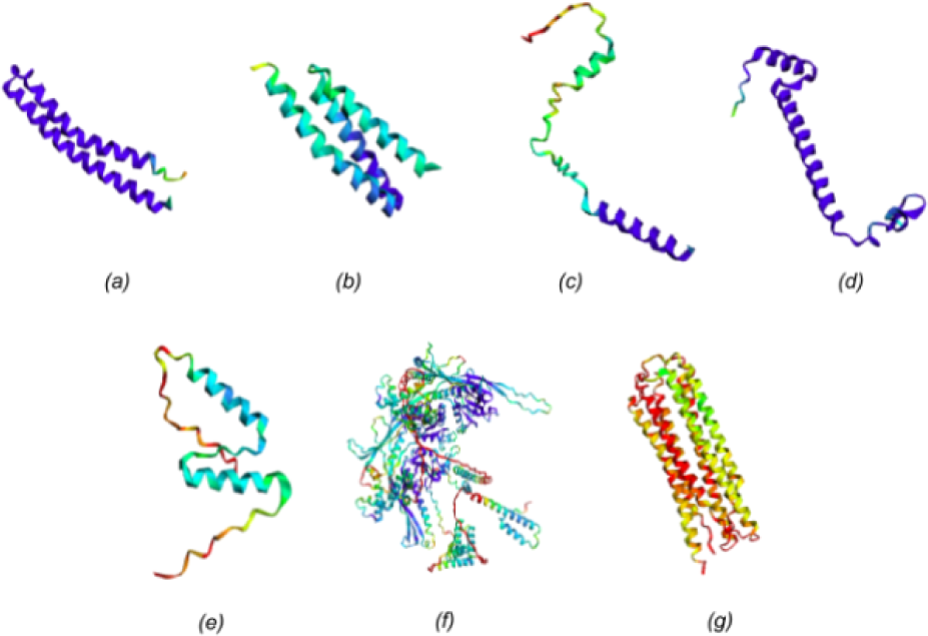
ColabFold predictions analysis for *M. avium*, *M. tuberculosis* H37Rv, *M. bovis*, and *M. bovis* BCG str. Pasteur. The red region corresponds to pLDDT very low confidence values (<50). The yellow region corresponds to pLDDT low confidence values (60). The green region corresponds to pLDDT ok confidence values (70). The light blue region corresponds to pLDDT confidence values (80). The dark blue region corresponds to pLDDT very high confidence values (>90). (a) *M. avium* MAP01 protein. (b) *M. bovis* BCG MBCGP02 protein. (c) *M. bovis* BCG MBCG03 protein. (d) *M. tuberculosis* H37Rv MHR04 protein. (e) *M. tuberculosis* H37Rv MHR05 protein. (f) *M. bovis* SP38 MSP06 protein. (g) *M. bovis* SP38 MSP07 protein.

Examination of the pLDDT plots derived from the prediction of *Mycobacterium* strains suggests the existence of high-confidence regions. All regions identified in the plots can be seen in the final model as a complex formation of alpha-helices (Figure 2). These helices compose the transmembrane part of the protein. The comparison between the ColabFold model with the corresponding structure of the online AlphaFold database produces a superposition with a TM-score of 0.74 and an RMSD of 2.57 Å. This deviation is caused purely by the misalignment of the low pLDDT regions. However, all the high-confidence regions are precisely aligned.

### Cloning, Transformation, Expression, and Purification of Recombinant Protein

**Figure 3:**
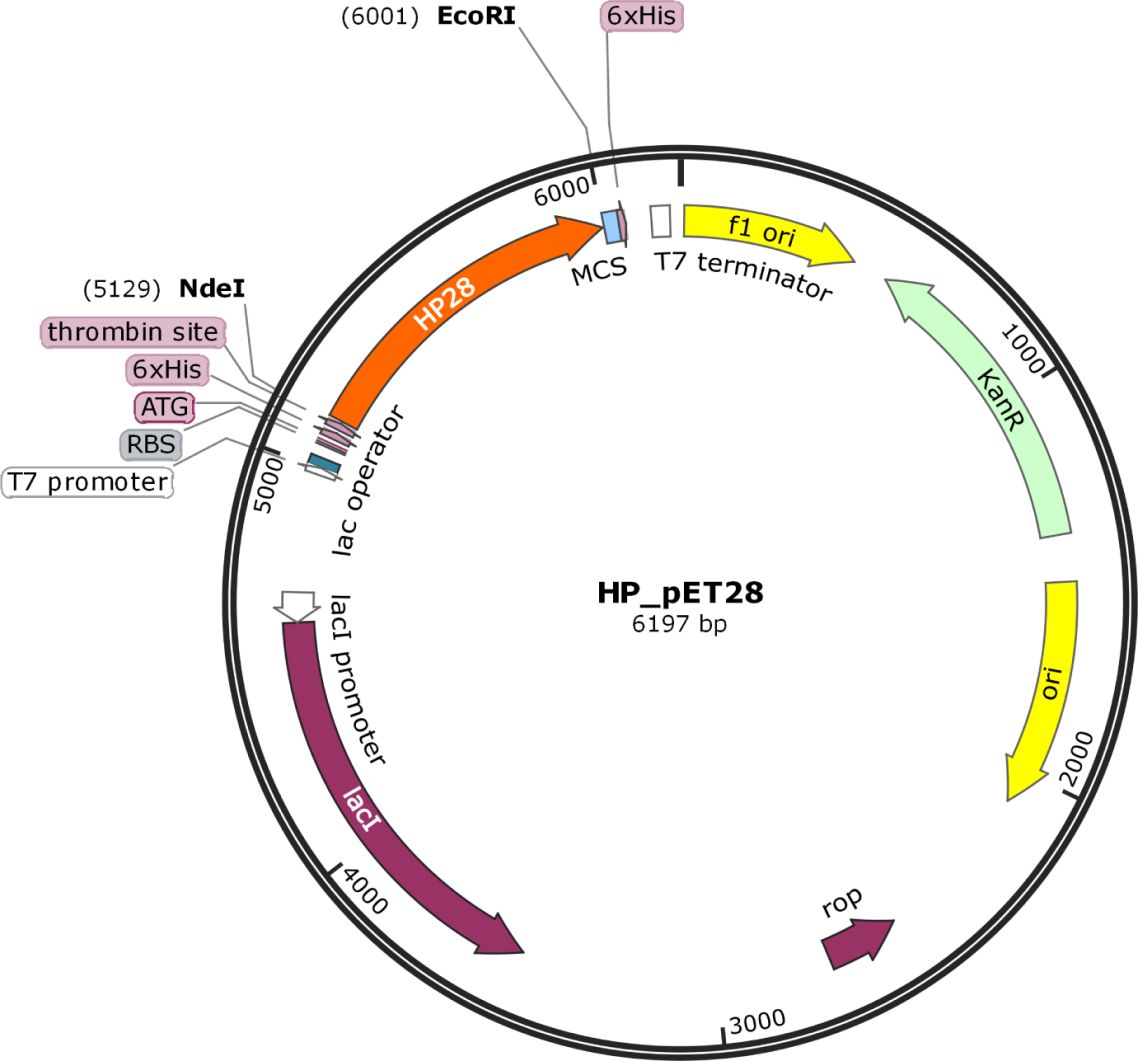
Construction map of the gene that origins the HP28 protein in plasmid pET28a, visualized in SnapGene 1.1.3 software.

The HP28 protein gene sequence was successfully cloned in the pET-28a vector (Figure 3).

**Figure 4:**
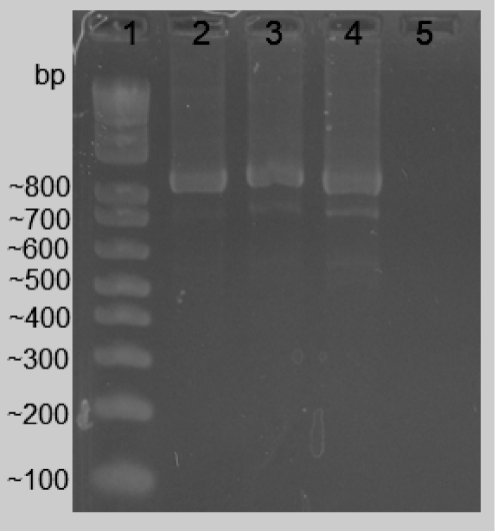
Electrophoresis of PCR reaction using primer T7 in agarose gel 2%. 1—Invitrogen 100 bp DNA Ladder. 2–4—Amplification of transformed colonies. 5—Negative control (colony not transformed).

Transformation in *E. coli* was confirmed by the formation of colonies in plates with kanamycin and by colony PCR using specific primers for the T7 promoter, which resulted in size amplification of 879 bp (Figure 4).

**Figure 5:**
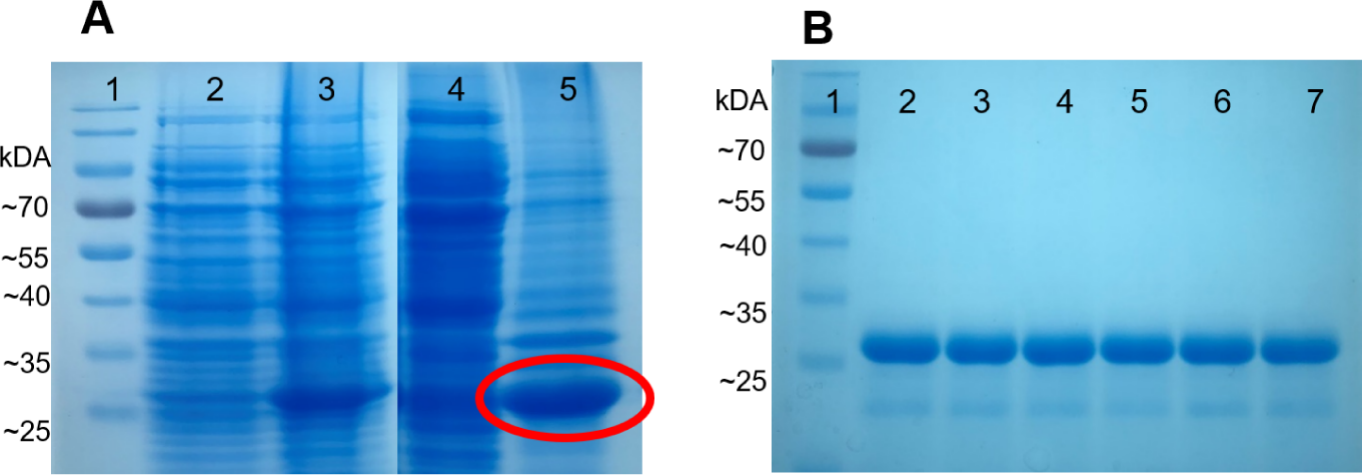
(A) SDS-PAGE 12.5% gel of protein expression. 1— PageRuler Prestained Protein Ladder molecular marker, 10 to 180 kDa; 2—Un-induced culture; 3—Culture induced with 1 mM IPTG; 4—Soluble Fraction; 5—Insoluble Fraction. (B) SDS-PAGE of protein purification. 1—PageRuler Prestained Protein Ladder molecular marker, 10 to 180 kDa; 2–7—Fractions of rHP28 protein eluted during purification.

Comparative analysis of the pre- and post-induction extract showed the presence of a band with the expected size of 30 kDa, indicating efficient production of the protein of interest. To determine in which fraction the protein was expressed, soluble and insoluble fractions collected during the expression process were analyzed by SDS-PAGE (Figure 5A). After protein purification and dialysis, the presence of the protein of interest was observed on the gel (Figure 5B). Protein quantity was measured using micro-BCA, and the production yield was 34 mg/L.

### Intradermal Skin Test

The delayed-type hypersensitivity response was evaluated in vivo using guinea pigs. None of the animals exhibited cutaneous reactions or any other signs associated with the saline solution injections.

**Figure 6:**
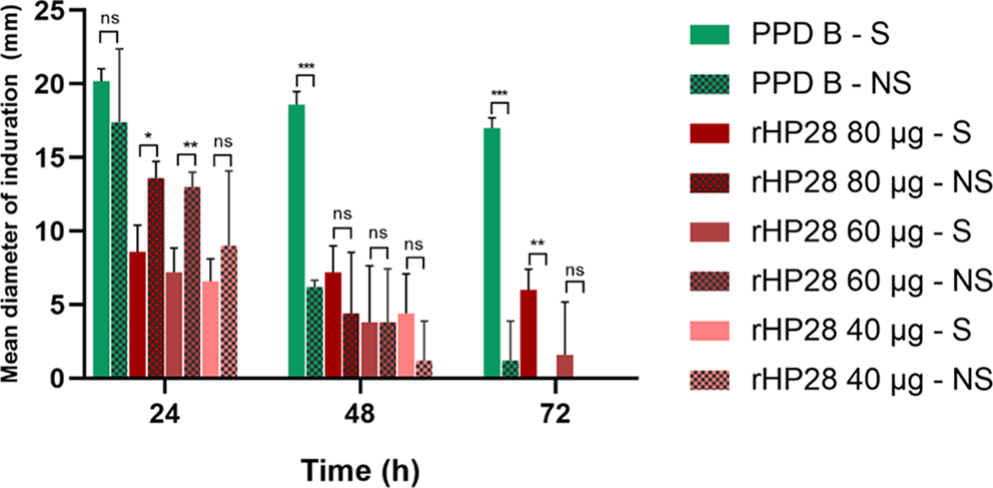
Analysis of intradermal skin test for PPD-B and rHP28 antigens in sensitized (S) and non-sensitized (NS) animals. Error bars demonstrate the standard deviations (SD). Statistical significance was determined using ANOVA *, **, and *** indicate significant differences between groups at *p* < 0.05, *p* < 0.005, and *p* < 0.0001, respectively; ns indicates non-significance.

In accordance with Brazilian Technical Regulations, animals are considered positive when they exhibit a reaction greater than 4 mm after 72 h post-injection.^17^ In this study, induration was observed at all three measurement intervals, with PPD-B showing the highest reading, approximately 21 mm (Figure 6). The response to the rHP28 protein varied with concentration: at 40 μg/100 μL, the average diameters were 6.6 mm at 24 h and 4.4 mm at 48 h, with the reaction disappearing by 72 h. At 60 μg, rHP28 produced diameters of 7.2 mm at 24 h and 3.8 mm at 48 h. Injection of 80 μg resulted in average diameters of 8.6 mm and 7.2 mm, which decreased to 6 mm across the three measurement intervals. The control group (NS) showed an initial reaction at 24 h for all antigens, which decreased or disappeared in subsequent readings.

A two-way ANOVA multiple comparisons test was conducted to assess the statistical difference between sensitized and non-sensitized groups. The results indicate that the rHP28 protein at 80 μg effectively distinguishes sensitized animals from non-sensitized ones (*p* < 0.006), with a 6 mm difference in reaction at the 72 h reading. Additionally, in the non-sensitized group at 72 h, the rHP28 protein showed no noticeable reaction, unlike PPD-B, which still produced a detectable, though weak, reaction.

## Discussion

The diagnosis of bovine tuberculosis currently faces challenges due to the lack of specific antigens for pathogenic bacteria, with the existing PPD antigen presenting limitations in both specificity and production. To address this issue, there is an ongoing search for novel targets that could replace PPD in the TST methodology.^14,18^ In this study, we present a comprehensive analysis of selected *Mycobacterium* strains, including their pangenomes and genomic islands. The focus was on identifying unique genes in each strain, particularly those from pathogenic *M. bovis*, to produce and test new antigens. Our bioinformatic analysis led to the identification of two unique genes, MSP06 and MSP07, from *M. bovis* SP38. We successfully produced one of these proteins and tested it as a TST antigen in guinea pigs. This approach has the potential to improve disease management in the livestock production industry.

Four *Mycobacterium* genomes of interest were chosen for this study. Among these, *M. bovis* SP38, the first strain isolated in Brazil, was chosen due to its relevance in developing new diagnostic methods to control bovine tuberculosis (bTB) in the country. This strain is particularly significant given the high prevalence of bTB in Brazil, coupled with widespread underreporting.^19^*M. tuberculosis* H37Rv was selected because it is the reference strain in most *in silico* and *in vitro* studies; and *M. bovis* BCG strain Pasteur was chosen to serve as a reference from a vaccine strain. Finally, the *M. avium* strain was integrated for comparison.

The dissection of the *Mycobacterium* pangenome into four different gene categories (“core”, “softcore”, “shell,” and “cloud”) can facilitate strategies for vaccine and the choice of the diagnostic target. Our results revealed variable numbers of cloud genes depending on the strain evaluated, and 12 were identified for *M. bovis* SP38, giving us different possibilities of unique genes to explore. The use of pangenome analysis has already been reported for several organisms, including *Enterococcus faecium*,^20^ Epstein-Barr virus,^21^*Leptospira*,^22^*Mycobacteroides abscessus*,^23^ Salmonella *enterica,*(24) and *Bordetella pertussis*.^25^ Moreover, our results also indicated that the *Mycobacterium* pangenome is open and the number of accessory genes will continue to increase, which aligns with the conclusions of other studies.^26,27^

An important goal in pathogen genomics is to identify and characterize genomic islands (GIs) in pathogenic microorganisms since certain virulence factors are strongly associated with GIs.^28^ Virulence factors are associated with horizontal gene transitions between species. As for *Mycobacterium*, ancient genomic islands from *M. tuberculosis* may be rooted to the pool of mobile genetic vectors distributed among *Mycobacteria*, as observed by another genomic island study.^29^ In this study, genomic islands (GIs) were identified for each genome, with the highest numbers found in *M. tuberculosis* and *M. bovis* and the lowest in *M. avium*. For diagnostic and vaccine development, it is crucial that the identified proteins are associated with pathogenicity and/or virulence, as they are more likely to elicit an immunogenic response in the host.^30^

Our objective of finding unique proteins for each strain was successful when combining the pangenome results with the GIs, considering alignments of less than 60% as the previously indicated threshold.^31^ The analysis generated one protein for *M. avium* and two proteins for *M. tuberculosis* H37Rv, *M. bovis*, and *M. bovis* BCG. Antigenicity analysis was also performed since the goal of this study was to test a protein for bTB diagnosis. From the two proteins identified for *M. bovis*, MSP07 (named HP28) showed an antigenicity score of 0.9272. Therefore, it was chosen for further cloning and expression. All sequences were submitted to ColabFold for protein structure visualization. The resulting models revealed that all proteins were composed of alpha-helices, the most common structural motif in transmembrane proteins.^32^ These proteins are considered suitable targets for therapeutics,^33^ alongside secreted and cell surface membrane proteins.^34,35^ An example of a widely studied alpha-helix protein, known for its virulence and potential as a vaccine or diagnostic candidate, is the ESAT-6 protein.^36^ A portion of the HP28 protein sequence has been identified in the literature as originating from the ESAT-6 family (UNIPROT accession number: P64094) and is excreted by the bacterium. The protein was selected for recombinant expression. Protein production was successfully obtained with a yield of 34 mg/mL. For *in vivo* testing, we estimated the initial protein concentration for *M. tuberculosis* according to studies previously conducted by our group,^37,38^ thus evaluating concentrations of recombinant protein ranging from 0.0004 to 0.04 μg per 100 μL. The injection point with 0.04 μg proved to be the formulation with the most efficient response, and for this reason, it was selected as our starting point. The ideal protein concentration in the standardization of an intradermal test is variable, and concentrations from 10 to 400 μg have already been reported.^39−41^ Here, we started at 40 μg, followed by 60 and 80 μg. Their response in the group sensitized with *M. bovis* (S) varied from 8.6 to 1.6 mm. Using 80 μg, rHP28 showed a desirable reaction response, within the threshold that differentiate positive from negative tests.^17^ We could also observe significant differences in response magnitude between PPD-B and recombinant proteins, which has also been reported in previous studies. Consequently, lower cutoff points should be considered when evaluating tests based on recombinant proteins or peptides.^42,43^

The most significant reaction in terms of diameter occurred when using 80 μg, reaching 6.6 mm. Three reading times were evaluated here. At the 24-hour period, non-sensitized animals exhibited a significant higher response than sensitized animals. This may be attributed to an initial innate immune response or nonspecific skin irritation in non-sensitized animals. In contrast, sensitized animals, previously exposed to the antigen, likely exhibited a more regulated immune response, with reduced reactivity due to tolerance or diminished inflammation resulting from prior exposure.^44^

Injections of 40 and 60 μg resulted in reactions of 7.2 mm and 6.6 mm, respectively, which decreased over time to 1.6 mm and 0 mm at the final reading. This reduction in reaction size between 24 and 48 h is commonly reported.^45^ During the 72 h period, only the 80-μg injection maintained a reaction size close to the initial 24 h measurement. As mentioned, initial reaction at 24 h was observed for all antigens in both groups, but it completely disappeared in the control group (NS), suggesting that a 24 h reading may not be recommended. Across all responses, the 72 h reading proved to be more accurate, as the PPD-B response was lower (2 mm) in non-sensitized animals. The protein tested here for the first time showed an even lower response, with no perceptible reaction in control animals, clearly differentiating between sensitized and non-sensitized groups. The 72 h induration reaction reading is also recommended by the bTB diagnostic manual^7^ and by Brazilian PNCEBT regulations.^17^ Additionally, readings taken after 72 h have been described in studies as ideal, as recombinant proteins such as ESAT-6 and CFP10 reach their peak reaction during this period.^40,41^

The ability of the rHP28 protein to elicit an induration response and distinguish between sensitized and non-sensitized animals suggests its potential use in bovine tuberculosis diagnosis. To our knowledge, this is the first study to identify and evaluate MSP07 (HP28) as a diagnostic antigen for bovine tuberculosis. This novel finding provides an important contribution to the field, as it demonstrates significant potential to improve diagnostic accuracy. Therefore, we propose its inclusion in diagnostic cocktails alongside ESAT-6, CFP10, and/or EsxI, for example, as antigens combined have been reported to enhance the host’s immune response, improve induration reactions, and consequently increase disease detection accuracy.^37,42^

## Conclusion

The bioinformatic tools used in this study provided valuable insights into the genetic composition and potential virulence factors of these *Mycobacterium* species. Additionally, these tools demonstrated significant potential in identifying immunogenic targets for the development and improvement of diagnostic tests, applicable not only to bovine tuberculosis but also to a variety of other infectious diseases. Moreover, the rHP28 protein was successfully cloned and expressed in *E. coli*. When tested intradermally in guinea pigs, the protein effectively differentiated between sensitized and non-sensitized animals, indicating its potential as an antigen for tuberculin skin tests (TST).

## Materials and Methods

**Figure 7:**
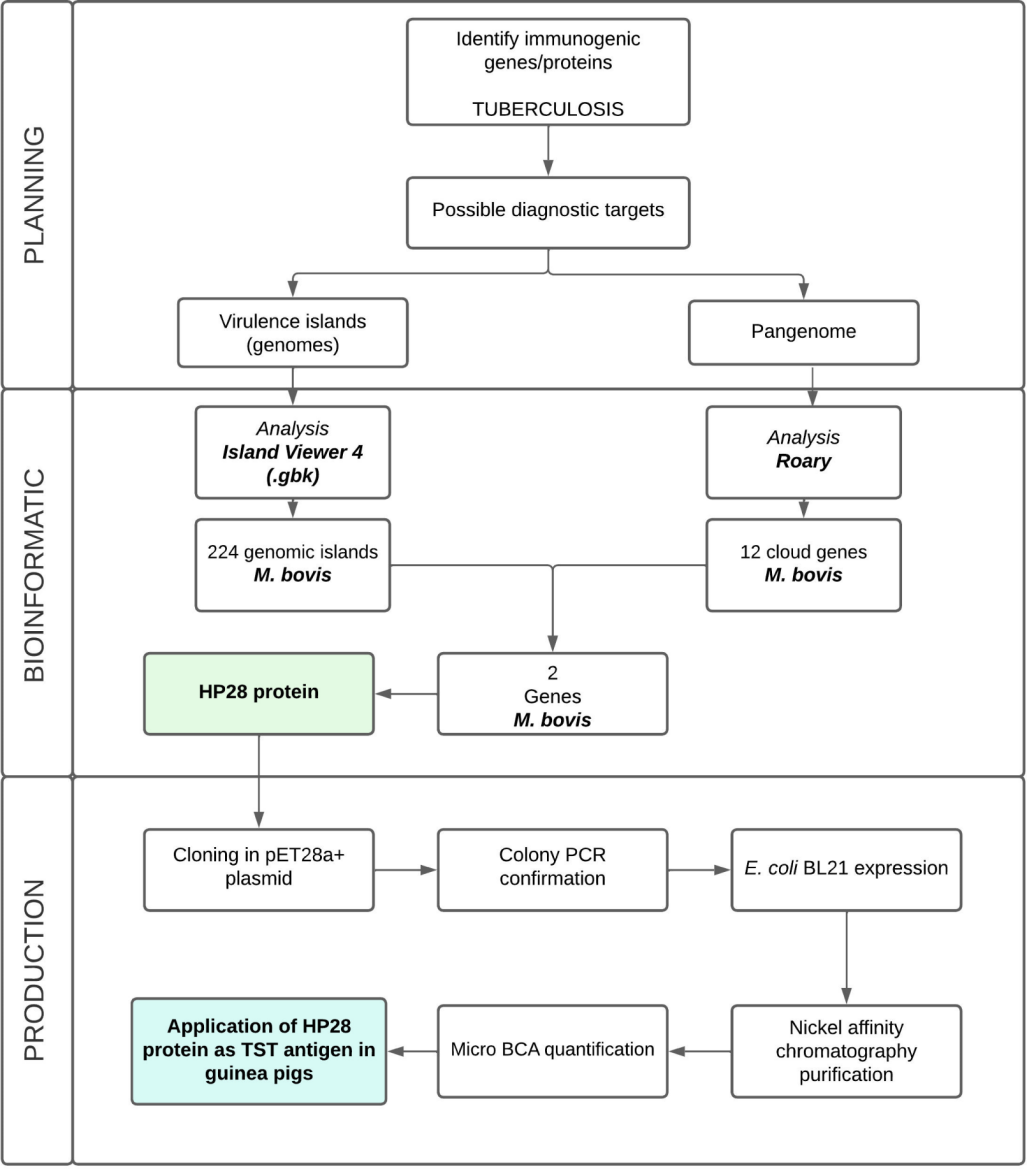
Overall workflow on the study, which was divided into three steps. The first phase consisted of developing the work plan; the second phase consisted of carrying out the bioinformatic study; the third and last phase consisted of testing the protein selected as the antigen to be used by intradermal reaction.

The project planning phase involved identifying novel targets for bovine respiratory diseases using two distinct approaches: (1) identifying unique genes through pangenome analysis and (2) detecting genomic islands associated with virulence. This was followed by the production and testing of the recombinant protein HP28. The workflow can be visualized in Figure 7. Detailed methodology is further described:

### Pangenome Analysis

The GFF3 genome files of four *Mycobacterium* strains (*M. bovis* SP38, *M. bovis* BCG, *M. bovis*, and *M. tuberculosis* H37Rv) were retrieved from the National Center for Biotechnology Information (NCBI) database (Table 1). To investigate homologous protein clusters across these genomes, Roary v3.13.0 was used to cluster orthologous genes (OGs) based on a minimum percentage identity of 90% with BLASTp.^46^ The analysis categorized genes into three types: core genes present in all strains, genes present in at least two strains, and strain-specific genes. A binary matrix representing the presence or absence of each gene across all strains was used to estimate the sizes of the pan-genome and core-genome using PanGP v1.0.1 with the totally random sampling algorithm.^46^ Gene distribution analysis was conducted using a Python script, which utilized the Roary gene_presence_absence.csv output files to perform genomic association analysis.

### Genomic Island Analysis

The software IslandViewer 4^50^ was used to predict GIs in the four strains of *Mycobacterium* selected (Table 2). GIs < 10 kb were considered genomic islands unless otherwise stated. Some GIs predicted by SIGI-HMM^51^ and IslandPath-DIMOB,^52^ two components of IslandViewer 4, were partially superimposed, increasing the number of predictions. After the identification of each predicted GI by IslandViewer 4, the regions were selected and visualized using Artemis software to generate .GBK and .FASTA files for each GI.^53^ These files contained the amino acid sequence, the name, and the protein ID of each product of each GI, and were used to create multi-fasta files for each microorganism.

**Table 2:** *Mycobacterium* Genomes Used in this Study and their Respective References

Name	Genome accession number	Reference
*Mycobacterium bovis* SP38	NZ_CP015773	Guimarães et al., 2015^47^
*Mycobacterium bovis* BCG Pasteur	NC_008769	Brosch et al., 2007^48^
*Mycobacterium avium* subsp. *paratuberculosis*	NZ_CP033910	Unpublished
*Mycobacterium tuberculosis* H37Rv	AL123456	Cole et al., 1998^49^

### Single Protein Analysis and Antigenicity Evaluation

To analyze the individual protein present in each strain, a database of SwissProt bacterial proteomes was downloaded from the UniProt database using a bash script. The output from the genomic island analysis was aligned with the bacterial proteome database through multiple alignments using the BLAST tool in Python. E-value parameters of 1e-50 and BLASTp for alignment were applied. Alignments with an identity of less than 60% were selected to identify unique proteins in each microorganism.

Specifically, for *M. bovis* pathogenicity, the pangenome and virulence island results were compared using a Python script to identify any unique genes within virulence genomic islands. An additional BLAST alignment was performed to ensure that the proteins were not similar to any other *Mycobacterium* species. The protein sequences were then submitted to antigenicity evaluation using ANTIGENPro (Scratch Protein), where a threshold of 0.5 was considered indicative of an antigenic protein, following the standard criterion established by the software.^54^

### Protein Structures

The open-source software ColabFold^55^ was used for the computational analysis of protein structures (versions 1.3.0 and 1.5.2). The notebook “ColabFold: AlphaFold2 using MMseqs2” was used through the Google Collaboratory platform to run AlphaFold with the following parameters:

*use_templates = false, use_amber = true, msa_mode = “MMseqs2 (UniRef + Environmental)”, model_type = “AlphaFold2-ptm”, num_models = 5, model_order = [1, 2, 3, 4, 5], num_recycles = 6, rank_by = “plddt,” max_msa = null, pair_mode = “unpaired + paired”.*

The wild-type sequences of the seven proteins of interest identified in step 3 (MAP01, MBP02, MBP03, MHP04, MHP05, MSP06, and MSP07) were submitted to ColabFold for structure prediction using the specified parameters. The predictions were made without the use of templates. The AlphaFold algorithm generated five structural models for each input, ranking them based on the overall predicted Local Distance Difference Test (pLDDT), AlphaFold’s per-residue confidence metric. Additionally, the Predicted Aligned Error (PAE) of each model was visualized in a matrix, indicating confidence in the relative positioning of residue pairs. All structural models underwent relaxation within the Amber99sb force field^56^ as part of the AlphaFold framework. To enhance the accuracy of the results, the number of recycles was increased to six, doubling the default three cycles.

### Bacterial Strains

The *M. bovis* strain was obtained from the Reference Bacteria Collection for Sanitary Surveillance at the Oswaldo Cruz Foundation (FIOCRUZ), Brazil. The bacteria were cultured in glass tubes containing Lowenstein-Jensen solid medium for 3–4 weeks at 37 °C.

### Cloning, Transformation, Expression and Purification of Recombinant Protein

The full sequence of HP28 was cloned and inserted into the pET28a+ expression plasmid. The plasmid was then used to transform *E. coli* BL21(DE3) via heat shock, using 50 μL of competent bacteria and 2 μL of plasmid under the following conditions: 20 min at 4 °C, 45 seconds at 42 °C, and 5 min at 4 °C. Transformation was confirmed through colony PCR using specific primers targeting the T7 promoter.

To express the protein, a pre-inoculum of 20 mL Terrific Broth (TB) medium with 30 μg/mL kanamycin was incubated overnight at 150 rpm and 37 °C. For the inoculum, 10 mL of the previous culture was transferred to 1 L of fresh TB medium, and the optical density (O.D.) was monitored. When the O.D. reached 0.5, the culture was induced with 1 mM IPTG and incubated overnight with agitation at 16 °C. The cells were harvested by centrifugation at 5000 g for 20 min, and the resulting pellets were sonicated in lysis buffer (50 mM Tris pH 8 and 50 μg/mL lysozyme). Following a second centrifugation under the same conditions, the pellet was subjected to further sonication in urea buffer (50 mM Tris, 300 mM NaCl, 10 mM imidazole, and 8 M urea). After another centrifugation cycle, the supernatant (insoluble fraction) was collected for purification.

Protein purification was performed using HisTrap HP 5 mL Nickel affinity chromatography column (Cytiva). The column was equilibrated with binding buffer (8 M urea, 10 mM imidazole, 300 mM NaCl, and 20 mM NaH_2_PO_4_, pH 7.2). After sample loading, 50 mL of washing buffer (8 M urea, 20 mM imidazole, 300 mM NaCl, and 20 mM NaH_2_PO_4_, pH 7.2) was applied to remove nonspecific proteins. For elution, 10 to 15 mL of elution buffer (8 M urea, 500 mM imidazole, 300 mM NaCl, and 20 mM NaH_2_PO_4_, pH 7.2) were collected in 2 mL fractions for electrophoresis analysis. After purification, the most concentrated protein aliquots were dialyzed to remove urea and imidazole. Buffer exchanges with 50 mM Tris/0.05% Triton X-100, pH 8.5, were performed every hour to gradually remove urea. Finally, the samples were evaluated by SDS-PAGE. Protein quantification was carried out using the Micro BCA Protein Assay Kit (Thermo Scientific), and *E. coli* LPS was removed using Pierce High-Capacity Endotoxin Removal Spin Columns (Thermo Scientific).

### Intradermal Skin Test

For the production of *M. bovis* inocula, 100 mg of wet mass from mycobacteria cultured on Löwenstein-Jensen solid medium was homogenized with 25 mL of sterilized mineral oil. The mixture was inactivated by autoclaving in flowing steam for 1 h. The final concentration of the prepared inoculum was 4 mg/mL.

The use of ten guinea pigs weighing between 250 and 350 g was approved by the Ethics Committee on Animal Use (CEUA) of the Instituto de Tecnologia do Paraná (TECPAR) under protocol 009/2022. Five female animals were sensitized by intramuscular injection of 0.5 mL (2 mg/mL) of inactivated *M. bovis* and were classified as the sensitized group (S). The remaining five animals served as the control group (NS – not sensitized to *M. bovis*). Both groups were observed for 30 days.

After the 30-day period, the guinea pigs were shaved at the intercostal region, and each antigen was injected intradermally in a volume of 100 μL. In addition to recombinant protein antigens, standard bovine PPD tuberculin (Laboratorios Microsules) was injected as a positive control, and pure diluent was used as a negative control. The reactions to the injections were assessed 24, 48, and 72 hours post-injection, based on the diameter of the palpable induration area. In Brazil, intradermal reactions are classified according to criteria defined in the Technical Regulation of the Ministry of Agriculture.^17^ Animals considered positive exhibit a reaction greater than 4 mm 72 h post-injection.

Statistical analysis of the experiment was conducted using GraphPad Prism 8.0 software.

## Supporting Information Available

Figure S1. Genomic islands of virulence and pathogenicity predicted by IslandViewer 4. The islands marked with a dark purple dot are genes for virulence factors. Light purple dots are homologous to virulence factors; dark pink dots are resistance genes; light pink dots are the homologous resistance genes, while the ones marked in yellow are pathogen-associated genes. (A) *M. avium*. (B) *M. tuberculosis* H37Rv. (C) *M. bovis* SP38. (D) *M. bovis* BCG str. Pasteur (PDF)

## Abbreviations

bTB: bovine tuberculosis
GI: genomic islands
kDa: kilodalton
Mtb: *Mycobacterium tuberculosis*
MTC: *M. tuberculosis* complex
NCBI: National Center for Biotechnology Information
OG: orthologous genes
PPD: purified protein derivative
TST: tuberculin skin test
