# Supplementary material for: Integrated Bioinformatics Analysis for Target Identification and Evaluation of Recombinant Protein as an Antigen for Intradermal Skin Test in Bovine Tuberculosis Diagnosis
Source: ACS Omega. 2025 Feb 24;10(9):9187–96. doi: 10.1021/acsomega.4c09374 (PMC11904847; doi:10.1021/acsomega.4c09374)
Supplement: Supplementary file 1 — ao4c09374_si_001.pdf [file ao4c09374_si_001.pdf]

Integrated Bioinformatics Analysis for Target Identification and Evaluation of Recombinant Protein as an Antigen for Intradermal Skin Test in Bovine Tuberculosis Diagnosis

Violetta Dias Pacce[1], Amanda Munari Guimarães[2], Frederico Schmitt Kremer[2], Gabriela Nascimento Ferreira[1], Jean Michel Dela Vedova-Costa[1], Aline Cristina dos Santos[3], Odir Antônio Dellagostin[2], Carlos Ricardo Soccol[1], Vanete Thomaz-Soccol[1*]

[1] Laboratório de Biologia Molecular, Programa de Pós Graduação em Engenharia de Bioprocessos e Biotecnologia, Universidade Federal do Paraná, Postal Code 81531-990, Curitiba - PR, Brazil

[2] Programa de Pós Graduação em Biotecnologia, Centro de Desenvolvimento Tecnológico, Universidade Federal de Pelotas, Postal Code 96160-000, Pelotas - RS, Brazil

[3] Laboratório Provas Biológicas, Instituto de Tecnologia do Paraná, Postal Code 80035-060, Curitiba - PR, Brazil

Supporting Information

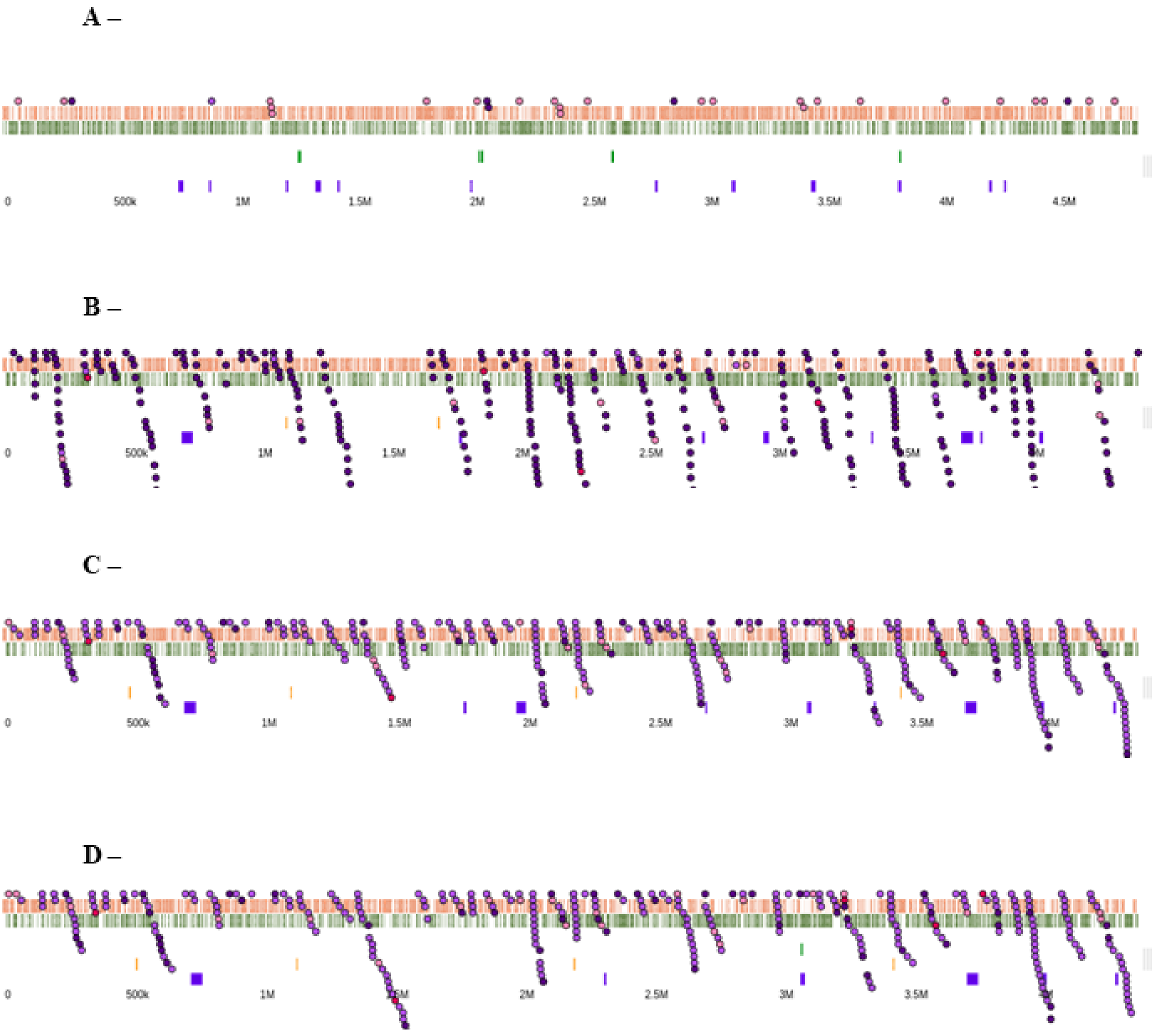

Figure S1. Genomic islands of virulence and pathogenicity predicted by IslandViewer 4. The islands marked with a dark purple dot are genes for virulence factors. Light purple dots are homologous to virulence factors; Dark pink dots are resistance genes; Light pink dots are the homologous resistance genes, while the ones marked in yellow are pathogen-associated genes. (A) *M. avium.* (B) *M. tuberculosis* H37Rv. (C) *M. bovis* SP38. (D) *M. bovis* BCG str. Pasteur.
